# Gender differences in non-motor fluctuations in Parkinson’s disease

**DOI:** 10.1007/s00702-023-02679-6

**Published:** 2023-08-01

**Authors:** Giulia Donzuso, Calogero Edoardo Cicero, Erica Vinciguerra, Rosy Sergi, Antonina Luca, Giovanni Mostile, Claudio Terravecchia, Mario Zappia, Alessandra Nicoletti

**Affiliations:** Department of Medical, Surgical Sciences and Advanced Technologies “GF Ingrassia”, University of Catania, Via Santa Sofia 78, 95123 Catania, Italy

**Keywords:** Parkinson’s disease, Non-motor symptoms, Non-motor fluctuations, Gender

## Abstract

Non-motor symptoms (NMS) and Non-motor fluctuations (NMF) in Parkinson’s Disease (PD) are common, involving several domains and affecting quality of life. Aim of the study is to estimate the burden of NMF in PD patients and to evaluate the possible gender effect. PD patients fulfilling the MDS-PD diagnostic criteria attending the “Parkinson’s Disease and Movement Disorders Centre” of the University of Catania were evaluated using the Non-Motor Fluctuations Assessment (NoMoFA) Questionnaire. NoMoFA items were also grouped into the following domains: cognitive, mood, sleep/fatigue, dysautonomia, hallucination/perception and miscellaneous domains were identified. One-hundred and twenty-one patients with PD (67 men, 55.4%; mean age 70.2 ± 8.9 years, disease duration 8.3 ± 4.6 years) were evaluated. All PD patients reported at least one NMS, whereas 87 (71.9%) also reported NMF. “Feel sluggish or had low energy levels” (47.2%) along with “Feel excessively sleepy during the day” (40.0%) were the most common NMF reported in the whole sample. The majority of PD patients reported the presence of NMF during the OFF state (79, 65.3%). At multivariate analysis, NMF were positively associated with the female gender (adjusted OR 3.13; 95%CI 1.21–8.11 *p*-value 0.01). Women with PD had higher NMF scores especially in depression/anxiety, sleep/fatigue and dysautonomia domains. Our study reported the presence of a gender-related pattern in the frequency of NMS and NMF in PD patients, with female gender associated with a higher risk of developing NMF, highlighting the need for personalized treatment strategies when addressing NMF.

**Supplementary Information:**

The online version contains supplementary material available at 10.1007/s00702-023-02679-6.

## Introduction

Despite the classic clinical motor features peculiar to the diagnosis (Postuma et al. 2015), it is well known that Parkinson’s disease (PD) is a complex disorder that encompasses also sensory abnormalities, behavioral and cognitive changes, sleep and gastro-intestinal disturbances, and autonomic dysfunction. Non-motor symptoms (NMS) are commonly experienced by PD patients and can appear at any time during the disease course leading to increased disability and affecting quality of life. Almost 100% of PD patients reported at least one NMS (Kim et al. 2013; Krishnan et al. 2011). 

Levodopa is the most effective symptomatic therapy for PD, but chronic levodopa therapy is associated with the development of motor complications, consisting of fluctuations in the motor response and involuntary movements or dyskinesias (Obeso et al. 2000). Nonetheless also NMS may fluctuate leading to the Non-Motor Fluctuations (NMF), the dynamic counterpart of NMS (Kleiner et al. 2021). NMF are reported by patients in both the ON and OFF phases, occurring in coincidence with motor fluctuations or independently, and represent one of the major determinants of disability and poor quality of life (Picillo et al. 2016). The mechanisms of NMF are not well understood. It is thought that they share dopaminergic mechanisms with motor complications, but probably involving also different neural pathways (Martínez-Fernández et al. 2016).

Gender differences have been acknowledged as important determinants of neurodegenerative diseases, including PD (Meoni et al. 2020). In particular, a higher incidence of PD and a more severe progression have been reported among men, while, as the disease progresses, women are at higher risk of developing highly disabling motor complications, such as motor fluctuations and dyskinesias (Picillo et al. 2017). Several studies have also assessed gender differences in NMS (Meoni et al. 2020; Picillo et al. 2017) reporting a higher prevalence of some NMS such as sadness, nervousness, constipation, restless legs and pain among women, while daytime sleepiness, dribbling saliva, reduced interest in sex seem to be more prevalent in men (Picillo et al. 2017). On the other hand, the evaluation of NMF is rarely part of clinical routine and little is known about the real burden of NMF. Recently, Kleiner and coll (Kleiner et al. 2021) validated a new questionnaire capturing the presence and severity of NMS and NMF in PD patients, the Non-Motor Fluctuation Assessment Questionnaire (NoMoFA), proposing a reliable and effective tool for both static and fluctuating symptoms (Kleiner et al. 2021).

Aim of this study is to estimate the frequency of static NMS and dynamic NMS (i.e. NMF) and gender differences in PD patients assessed by the NoMoFA questionnaire.

## Materials and methods

### Study population

Patients with a diagnosis of PD according to the Movement Disorder Society (MDS)-PD diagnostic criteria (Postuma et al. 2015) on dopaminergic treatment with levodopa, attending the “Parkinson’s Disease and Movement Disorders Centre” of the University of Catania were consecutively enrolled during their regular visits. All PD patients underwent a complete neurological examination and motor disability was evaluated according to the Unified Parkinson’s Disease Rating Scale-Motor Examination (UPDRS-ME) (Fahn and Elton 1987) and the Hoehn and Yahr (HY) scale (Hoehn and Yahr 1967). Presence of motor complications, motor fluctuations and dyskinesia, was assessed using items 32–39 of the UPDRS part IV. Pharmacological history was reported, and the Levodopa Equivalent Daily Dose (LEDD) was calculated (Tomlinson et al. 2010). According to our clinical routine, PD patients underwent clinical evaluation in “practical OFF state”, thus before taking the first daily dose of the dopaminergic drug after an overnight fast. The presence of atypical parkinsonism, drug-naïve PD patients as well as the treatment with dopamine-agonists or MAO-B inhibitors alone were considered exclusion criteria. The collection of data was approved by our local Ethical Committee (Code 316/2015).

### NMS and NMF assessment

For the NMS and NMF evaluation, NoMoFA Questionnaire was administered in the original paper format during routine clinical patient care without modifications First, the NoMoFA questionnaire was translated into Italian by two native Italian speakers, then it was back translated into English to compare with the original one. Comprehension of the items has been tested during a pilot study involving 20 patients who were not included in the present study. Then, the translated version was administered face-to-face by two well-trained neurologists to avoid misinterpretation. Briefly, NoMoFA Questionnaire includes 27 items about NMS and the patient is asked to answer whether the NMS is present and how bothersome it is (mild, moderate, or severe – 1–3 points). Then it is asked if the NMS is worse during ON or during OFF time or if there is no difference. The “total NMF subscore” is the sum of the subscores “NMF ON” and “NMF OFF”, while the total (static) NMS score is the sum of all “NMS no difference” scores. The total NoMoFA score is calculated using the formula: Total NoMoFA = Total NMF (ON + OFF) + Total (static) NMS.^5^ PD patients reporting at least one symptom in OFF or ON state were considered non-motor fluctuating patients. Administration of the questionnaire was made in “practical OFF” state.

Additionally, considering all the 27 items, the following domains have been identified: attention (items 1–4), memory (item 15), language (item 5), depression/anxiety (items 6–10), impulsiveness (items 12–14 and 16), apathy (item 17), hallucination/perception (items 11 and 23), sleep/fatigue (items 18–19), dysautonomia (including cardiovascular, urinary and gastrointestinal function, items 24–27) and miscellaneous (pain, short of breath, items 20–22).

### Statistical analysis

Data were analyzed using STATA 16 software packages (StataCorp, College Station, TX, USA). Quantitative variables were described using mean and standard deviation. Qualitative variables were expressed as number and percentage. Differences between means and proportions were evaluated and t-test and the chi-square test, respectively. Normal distribution was assessed using the Shapiro–Wilk test and, in case of a not normal distribution, appropriate non-parametric tests were performed. When needed, variables have been dichotomized according to the median value of the pooled distribution.

Univariate logistic regression analysis was performed to evaluate possible associations between gender and NMF (outcome of the study) and demographic and clinical characteristics. Multivariate logistic regression analysis was performed adjusting for age and gender considered as a priori confounders and for each variable associated with the outcome at univariate analysis with a threshold of *p*-value = 0.10. The model was manually constructed using the likelihood ratio test (LRT) to compare the log-likelihood of the model with and without a specific variable. The significance level was set at 0.05 and the 95% confidence intervals (CI) were calculated. Correction for multiple comparisons according to false discovery rate (FDR) was also applied. Finally, in order to compare our results to the original validation study of the NoMoFA scale, in agreement with the original study (Kleiner et al. 2021), the internal consistency has been evaluated using Cronbach’s alpha considering only the total NoMoFA score.

## Results

### Demographics and clinical characteristics

One-hundred and twenty-one patients with PD were consecutively enrolled (67 men, 55.4%; mean age 70.2 ± 8.9 years; disease duration 8.3 ± 4.6 years; UPDRS-ME score 36.5 ± 13.6). Out of the 121 patients according to items 32–39 of the UPDRS part IV, 67 (55.4%) had motor complications, of whom 57 (47.1%) motor fluctuations, and 42 (34.7%) dyskinesias. There were no differences between men and women with PD in demographic and clinical characteristics, except for the presence of motor complications (*p-value* 0.03), motor fluctuations (*p-value* 0.04) and use of benzodiazepines (*p-value* 0.02) (Table 1). At univariate analysis, LEDD > 500 mg, female gender, age at onset and disease duration were significantly associated with the presence of motor complications as shown in supplementary table 1. At multivariate analysis, a significantly stronger positive association was found with LEDD and female gender (LEDD > 500 mg daily AdjOR 5.12; 95%CI 1.91–13.7; *p-value* 0.001; female gender AdjOR 3.48; 95%CI 1.45–8.39; *p-value* 0.004), while a significant negative association was found between age and presence of motor complications (Adj OR 0.97; 95%CI 0.90–0.99; *p-value* 0.03) (Supplementary table 1).

**Table 1:** Demographics and clinical characteristics of the whole PD sample and stratified by gender

	PD sample *N* = 121	PD men *N* = 67	PD women *N* = 54	OR	95%CI	*P* value
Age (years)	70.2 ± 8.9	70.2 ± 8.3	70.2 ± 9.7	0.99	0.96–1.04	0.9
Disease duration (years)	8.3 ± 4.6	8.5 ± 5.0	8.0 ± 4.2	0.97	0.90–1.05	0.5
UPDRS-ME score (OFF)	36.5 ± 13.6	37.8 ± 14.3	34.8 ± 12.7	0.98	0.95–1.01	0.2
Hoehn – Yahr stage	2.4 ± 0.8	2.4 ± 0.8	2.4 ± 0.8	0.93	0.59–1.47	0.8
LEDD (mg)	593.6 ± 362.6	648.8 ± 418.8	525.3 ± 266.0	0.99	0.99–1.00	0.06
Motor complications, *n* (%)	67 (55.4)	31 (46.3)	36 (66.7)	2.32	1.10–4.88	**0.03**
*Motor fluctuations, n (%)*	57 (47.1)	26 (38.8)	31 (57.4)	2.12	1.02–4.40	**0.04**
*Dyskinesia, n (%)*	42 (34.7)	24 (35.8)	18 (33.3)	0.89	0.42–1.90	0.8
iCOMT, *n* (%)	14 (11.6)	13 (19.4)	1 (1.8)	0.34	0.11–1.07	0.06
Antidepressants, *n* (%)	32 (32.2)	19 (28.4)	20 (37.0)	1.48	0.69–3.19	0.3
Benzodiazepines, *n* (%)	33 (27.3)	24 (35.8)	9 (16.7)	0.35	0.14–0.85	**0.02**
Antipsychotics, *n* (%)	14 (11.6)	9 (13.4)	5 (9.2)	0.65	0.20–2.09	0.5
*NoMoFA Questionnaire*						
Non-Motor Fluctuation, *n* (%)	87 (71.9)	41 (61.2)	46 (85.2)	3.64	1.48–8.94	**0.005**
Total symptoms	12.6 ± 4.9	13.0 ± 4.6	12.0 ± 5.1	0.24	0.88–1.03	0.2
Total NMF	5.5 ± 6.4	3.5 ± 4.8	7.9 ± 7.2	0.92	0.88–0.96	**0.001**
Total (static) NMS	15.0 ± 10.0	17.9 ± 10.1	11.3 ± 8.8	0.97	0.94–1.01	**0.005**
Total NoMoFA	20.5 ± 10.3	21.5 ± 9.9	19.2 ± 10.8	0.97	0.94–1.01	0.2

Data are given as means ± standard deviation or frequencies (%). *PD* Parkinson’s Disease, *UPDRS-ME* Unified Parkinson’s Disease Rating Scale-Motor Exam, *LEDD* Levodopa equivalent daily dose, *iCOMT* catechol-O-methyltransferase inhibitor, *NoMoFA* Non-motor Fluctuation Assessment, *NMF* non-motor fluctuations, *NMS* non-motor symptoms

### NMSs and NMF

All 121 enrolled PD patients (100%) reported at least one NMS, while 87 reported NMF leading to a prevalence of 71.9% (95% CI 63.1–79.8). The mean total NoMoFA score was 20.5 ± 10.3 without significant differences between men and women (Table 1), conversely the mean total NMF score was significantly higher among women (7.9 ± 7.2 *versus* 3.5 ± 4.8; *p-value* 0.001), while the mean total (static) NMS score was significantly higher among men (17.9 ± 10.1 *versus* 11.3 ± 8.8; *p-value* 0.005) as shown in Table 1.

Concerning the internal consistency, based on the total NoMoFA score, we obtained a Cronbach’s alpha of 0.84.

**Fig. 1:**
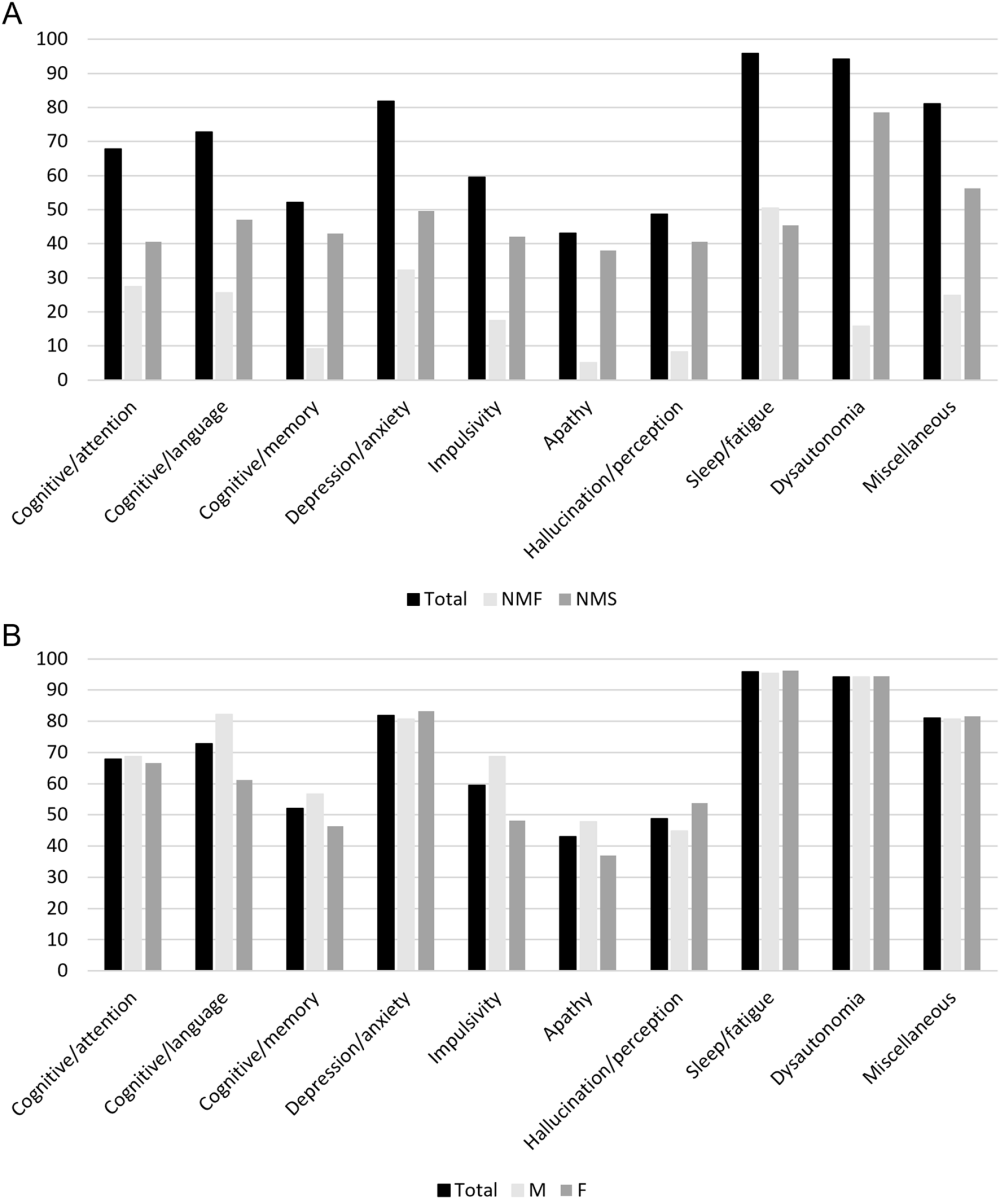
Frequency of reported domains in PD sample. **A** differences in reported non-motor symptoms in the whole PD sample considering non-motor fluctuation and static non-motor symptoms. **B** differences in reported domains between PD male and female. *PD* Parkinson’s disease, *M* male, *F* female

Regardless of the presence of fluctuations, “Feel sluggish or had low energy,” was the most common NMS reported by 87.6% of patients, followed by “Feel excessively sleepy during the day” (78.5%) and “Difficulty finding the right words when speaking” (71.9%) (Table 2), without significant differences between men and women with PD in all the reported items (Supplementary Table 2). Considering the NMS domains, sleep/fatigue was the most common (95.8%), followed by dysautonomia (94.2%), depression/anxiety (81.8%) and miscellaneous (81.0%) (Fig. 1). Men with PD showed a higher frequency of cognitive, sleep/fatigue and mood (depression/anxiety, impulsivity and apathy) static NMS compared to women (Supplementary Table 3).

**Table 2:** Frequencies of reported non-motor symptoms considering static and fluctuating symptoms

NMS	NMS *n* (%)	NMF *n* (%)	Static NMS *n* (%)
1. Lose your train of thought	65 (53.7)	21 (32.3)	44 (67.7)
2. Get distracted from completing a task	62 (51.2)	17 (27.4)	45 (72.6)
3. Difficulty planning or carrying out an activity	47 (38.8)	18 (38.3)	29 (61.7)
4. Confused such that you had difficulty performing simple tasks	40 (33.1)	13 (32.5)	27 (67.5)
5. Difficulty finding the right words when speaking	87 (71.9)	31 (35.6)	56 (64.4)
6. Excessively worried	79 (65.3)	21 (26.6)	58 (73.4)
7. Feel scared or threatened	20 (16.5)	6 (30.0)	14 (70.0)
8. Feel restless	76 (62.8)	24 (31.6)	52 (68.4)
9. Feel hopeless or excessively sad	76 (62.8)	23 (30.3)	53 (69.7)
10. Feel lonely or isolated	32 (26.4)	4 (12.5)	28 (87.5)
11. See things or people that were not there	31 (25.6)	6 (19.3)	25 (80.7)
12. Make poor decisions	26 (21.5)	2 (7.8)	24 (92.2)
13. Act quickly without thinking things through	31 (25.6)	7 (22.6)	24 (77.4)
14. Have a strong uncontrollable urge to do things	17 (14.0)	3 (17.6)	14 (82.4)
15. Have poor short-term memory	61 (50.4)	10 (16.4)	51 (83.6)
16. Have difficulty handling stressful situations	48 (39.7)	10 (20.8)	38 (79.2)
17. Lose interest in activities that you previously enjoyed	50 (41.3)	5 (10.0)	45 (90.0)
18. Feel sluggish or had low energy levels	106 (87.6)	50 (47.2)	56 (52.8)
19. Feel excessively sleepy during the day	95 (78.5)	38 (40.0)	57 (60.0)
20. Have painful sensations in your body	68 (56.2)	16 (23.5)	52 (76.5)
21. Have strange sensations in your body	63 (52.1)	17 (27.0)	46 (73.0)
22. Feel short of breath	54 (44.6)	12 (22.2)	42 (77.8)
23. Have problems with vision	50 (41.3)	3 (6.0)	47 (94.0)
24. Have excessive sweating	32 (26.4)	8 (25.0)	24 (75.0)
25. Feel that your heart was racing, had skipped a beat, or was pounding	35 (28.9)	7 (20.0)	28 (80.0)
26. Urinate more frequently or felt you had to go to the bathroom urgently	86 (71.1)	10 (11.6)	76 (88.4)
27. Have difficulty having a bowel movement	85 (70.2)	5 (5.9)	80 (94.1)

Data are given as frequencies (%). *PD* Parkinson’s Disease, *NMS* non-motor symptoms. *NMF* non-motor fluctuation

A total of 87 PD patients (71.9%) reported at least one non-motor symptom in OFF or ON state and then were considered as non-motor fluctuating (NMF +). There were no demographic or clinical differences between NMF + and NMF-, except for gender distribution (NMF + 46 women [85.2%] *versus* 41 men [38.8%]; *p-value* 0.005), Hoehn–Yahr stage and the presence of motor complications (motor fluctuations and/or dyskinesia), as shown in Table 3.

**Table 3:** Demographics and clinical characteristics of PD sample according to the presence of non-motor fluctuations

			*Univariate analysis*			*Multivariate analysis*		
	NMF + *N* = 87	NMF- *N* = 34	OR	95%CI	*P* value	AdjOR	95%CI	P value
Gender								
Male (Reference)	41 (38.8)	26 (61.2)	1	/	**/**	1	/	**/**
Female	46 (85.2)	8 (14.8)	3.64	1.48–8.94	**0.005**	3.13	1.21–8.11	0.01*
Age (years)	69.9 ± 9.5	71.0 ± 7.4	0.98	0.94–1.03	0.57	0.98	0.92–1.03	0.4
Disease duration (years)	8.5 ± 4.9	7.8 ± 4.1	1.03	0.94–1.13	0.42	/	/	/
UPDRS-ME score	37.5 ± 13.6	33.8 ± 13.6	1.02	0.99–1.05	0.18	/	/	/
Hoehn – Yahr stage	2.5 ± 0.8	2.2 ± 0.6	2.02	1.04–3.91	**0.04**	2.04	0.99–4.17	**0.05**^**§**^
LEDD (mg)	618.9 ± 369.6	528.8 ± 340.8	1.00	0.99–1.00	0.22	/	/	/
*Motor Complications, n (%)*	57 (65.5)	10 (29.4)	4.56	1.92–10.77	**0.001**	3.25	1.30–8.16	**0.01°**
Motor Fluctuations, *n* (%)	50 (57.5)	7 (20.6)	5.21	2.04–13.25	**0.001**	/	/	/
Dyskinesias, *n* (%)	37 (42.5)	5 (14.7)	4.29	1.52–12.14	**0.006**	/	/	/
iCOMT, *n* (%)	11 (12.6)	3 (8.8)	1.68	0.59–4.72	0.32	/	/	/
Antidepressants, *n* (%)	32 (36.8)	7 (20.6)	2.24	0.88–5.73	0.09	/	/	/
Benzodiazepines, *n* (%)	22 (25.3)	11 (32.3)	0.70	0.29–1.68	0.43	/	/	/
Antipsychotics, *n* (%)	10 (11.5)	4 (11.8)	0.97	0.28–3.34	0.96	/	/	/
*NoMoFA*								
Total symptoms	12.8 ± 4.7	11.8 ± 5.3	1.04	0.96–1.13	0.27	/	/	/
Total NMF	7.6 ± 6.4	/	**/**	**/**	**/**	**/**	**/**	**/**
Total (static) NMS	13.5 ± 9.7	18.8 ± 10.1	0.95	0.91–0.98	**0.01**	**/**	**/**	**/**
Total NoMoFA	21.2 ± 10.4	18.8 ± 10.1	1.02	0.98–1.06	0.25	/	/	/

Data are given as means ± standard deviation or frequencies (%). *PD* Parkinson’s Disease, *NMF* ±  PD patients with or without non-motor fluctuations, *UPDRS-ME* Unified Parkinson’s Disease Rating Scale-Motor Exam, *LEDD* Levodopa equivalent daily dose, *iCOMT* catechol-O-methyltransferase inhibitor, *NoMoFA* Non-motor Fluctuation Assessment, *NMF* non-motor fluctuations, *NMS* non-motor symptoms. *adjusted for age, Hoehn–Yahr stage and the presence of motor complications. ^§^adjusted for age, gender and the presence of motor complications. Adjusted for age, Hoehn–Yahr stage and gender

“Feel sluggish or had low energy levels” (47.2%) along with “Feel excessively sleepy during the day” (40.0%) were the most common NMF reported. Frequency of each single item according to the presence of fluctuations is reported in Table 2. The majority of PD patients reported the presence of NMF during the OFF phase (79, 65.3%) while 43 (35.5%) during the ON phase. The mean total ON score was 1.1 ± 2.2 while the mean total OFF was 4.3 ± 5.6. The most frequent NMF experienced in ON state were “Feel excessively sleepy during the day” (22.1%), “Act quickly without thinking things through” (19.4%), and “Have a strong uncontrollable urge to do things” (17.6%), whereas the most reported during OFF state were “Feel sluggish or had low energy levels” (36.8%), “Difficulty planning or carrying out an activity” (34.1%) and “Difficulty finding the right words when speaking” (33.3%). Concerning the different domains, the most frequently fluctuating domain (in OFF or ON phase) in the whole sample was sleep/fatigue (52.6%) followed by cognition (49.5%) when considering attention, memory and language as a unique domain. Depression/anxiety, apathy, sleep/fatigue, dysautonomia and miscellaneous were the fluctuating domains significantly more frequent among women with PD as shown in Supplementary Table 2.

NMF were reported by 61.2% of PD men and 85.2% of PD women, giving a crude OR of 3.64 (95%CI 1.48–8.94; *p-value* 0.005) at univariate analysis. A close association was found in multivariate analysis adjusting by age, Hoehn–Yahr stage and the presence of motor complications (adjusted OR 3.13; 95%CI 1.21–8.11 *p-value* 0.01) as shown in Table 3. Presence of NMF was also significantly associated with motor complications (motor fluctuations and/or dyskinesias) at both univariate and multivariate analysis (adjusting by Hoehn–Yahr stage, age and gender) with an adjusted OR of 3.25 (95%CI 1.30–8.16; *p-value* 0.01) as shown in Table 3. Nonetheless of the PD patients who reported NMF, 30 (34.5%) did not experience motor complications (motor fluctuations and/or dyskinesias) according to MDS-UPDRS part IV. Additionally, we found a significant positive association between NMF and HY stage at univariate analysis and borderline significant at multivariate analysis (AdjOR 2.04, 95%CI 0.99–4.17; *p-value* 0.05), while no association was found between NMF and UPDRS-ME, disease duration and LEDD (Table 3). In particular frequency of NMF (71.9%) was quite stable across the disease duration (0–5 years 72.9%, 6–10 years 67.2%, > 10 years 79.3%).

## Discussion

Our study confirmed a high frequency of NMS reported by all the enrolled PD patients but also demonstrated a high frequency of NMF recorded in 71.9% of our sample. Furthermore, our study highlighted a strong association between female gender and NMF. To the best of our knowledge, this is the first study aimed to evaluate the influence of gender on the risk of NMF in a cohort of PD patients using the new NoMoFA scale.

There is growing evidence that PD affects women and men differently and gender differences in epidemiology, clinical features and/or response to treatment have been reported. In particular, the male gender is associated with higher incidence, earlier disease onset, more severe motor symptoms and progression, and more frequent cognitive decline compared with the female gender (Meoni et al. 2020). However, as the disease progresses, women are at higher risk of developing highly disabling treatment-related complications, such as motor and non-motor fluctuations as well as dyskinesia, compared with men (Haaxma et al. 2007; Warren Olanow et al. 2013). Furthermore, several studies have also reported a “gender-effect” on NMS, both in treated (Picillo et al. 2014; Nicoletti et al. 2017) and drug-naïve PD patients (Picillo et al. 2013; Liu et al. 2015; Picillo and Fasano 2015). In agreement with the literature (Kleiner et al. 2021; Nicoletti et al. 2017; Rodriguez-Blazquez et al. 2020; Barone et al. 2009; Martinez-Martin et al. 2012), all subjects reported at least one NMS, and “*feeling sluggish or having low energy level*” was the most frequent one (Kleiner et al. 2021; Rodriguez-Blazquez et al. 2020). Although in our sample the overall frequency of NMS was similar between men and women, the (static) NMS total score was significantly higher among men with PD, and in agreement with the literature, we found a different gender-related static NMS distribution (Martinez-Martin et al. 2012; Solla et al. 2012). In particular, a higher frequency of cognitive disturbances and mood symptoms (including impulsivity and depression/anxiety) was reported by men with PD. Previous studies showed that male gender is one of the main risk factors for impulse control disorders development (Gatto and Aldinio 2019) and persistence (Aumann et al. 2020; Joutsa et al. 2012), thus we cannot exclude that impulsivity trait could be gender-related features independent from pharmacological treatment, considering also that in our sample there were no differences between men and women with PD concerning the use of dopamine agonists (19 men, 28.3% versus 16 women, 29.6%). Regarding the higher frequencies of benzodiazepines in men with PD (24 men, 35.8% *versus* 9 women, 16.7%, *p*-*value* 0.02), as previously demonstrated (Gaztanaga et al. 2021), it could be possible that in our sample the higher frequencies of NMS involving mood domain and anxiety among men could have led to increased use of this pharmacological therapy. Concerning the cognitive domain, our data are in agreement with literature findings (Nicoletti et al. 2017) showing the presence of a strong association between cognitive disturbances and PD men.

Nonetheless the main findings of our study concern the evaluation of the burden of NMF in a large sample of PD patients using the new NoMoFA scale, and the impact of gender on NMF.

It should be noted that even if it is well known that several NMS tend to develop fluctuations after chronic exposure to levodopa, data regarding the NMF are limited. Actually, despite the impact of NMF on quality of life, the subjective nature and the frequent lack of insight led to difficulties in NMF identification and quantification. Indeed, several validated questionnaires have been developed to evaluate the presence of NMS and some of these, such as the MDS-NMS Non-Motor Rating Scale (Chaudhuri et al. 2020), also include some specific items to detect non-motor OFF symptoms. Nonetheless none capture or quantify the entire spectrum of NMF in both the ON and the OFF-medication conditions (Kleiner et al. 2021). To the best of our knowledge, the NoMoFA is the first tool specifically developed to deeply assess the presence of NMF in both OFF and ON phase and it has been demonstrated to be valid and reliable in capturing both static and fluctuating NMS in PD (Kleiner et al. 2021).

In agreement with the literature data (Rodriguez-Blazquez et al. 2020), “*feeling sluggish or having little energy*” was the most fluctuating symptom in our cohort (47.2%). Gender-related difference in NMF was also found in several domains and in particular, depression/anxiety, apathy, sleep/fatigue, dysautonomia and miscellaneous were the non-motor fluctuating domains more frequently reported among women with PD. Furthermore, women also presented a significantly higher NMF total score. In our sample presence of NMF was strongly associated with the female gender with an Adjusted OR of 3.13. The higher frequency of NMF among women is in agreement with a previous observation reported by Picillo and coll (Picillo et al. 2016). In this latter study, in a cohort of de novo drug naïve PD patients followed up for four years, the female gender was associated with five times increased risk of developing NMF, evaluated by the means of the WOQ-19 questionnaire (AdjOR 5.33; 95%CI 1.21–23.4, *p*-*value* 0.03) (Picillo et al. 2016).

Although we have not a clear explanation for the higher frequency of NMF among women a possible role of sex hormones cannot be ruled out. As a matter of fact, preclinical studies have shown a possible neuroprotective role of estrogens and, according to literature evidence, lower levels are associated with a greater risk of developing motor fluctuations (Bovenzi et al. 2023).

According to a recent study, prevalence of NMF, evaluated by the means of the MDS-NMS rating scale (Chaudhuri et al. 2020), was low (9.1%) in the very early stage (< 2 years), showing a rapid increase between 2 and 5 years (54%) and a substantial plateau thereafter (van Wamelen et al. 2022). In our cohort prevalence of NMF (71.9%) was high and quite stable across the disease duration. However, it should be noted that in our sample only three PD patients presented a disease duration lower than 2 years (all reported at least one NMF), thus due to the low number of patients we are not able to evaluate the frequency of NMF in the very early stage of the disease. Furthermore, due to the use of different tools, comparison is limited.

Presence of NMF was mainly reported in OFF (65.3%) rather than in ON state (35.5%). It should be noted that the questionnaire was administered in OFF condition, thus we cannot exclude that ON or OFF states may also influence the perception of the NMS. In other words, we cannot exclude that NMS in ON state may be underestimated if recorded during the OFF phase or that may be underreported, as for dyskinesia, because perceived as a general greater well-being with respect to the OFF phase.

In our sample motor complication (presence of motor fluctuations and/or dyskinesia) were significantly positively associated with the NMF. As previously demonstrated (Brun et al. 2014; Seki et al. 2013), interestingly, not all patients complaining NMF were also complaining motor complications according to the UPDRS part IV, demonstrating that NMF may occur independently of the motor one. The occurrence of levodopa-related motor complications is dependent on several other factors and, as well known, the female gender is the most important one (Martínez-Fernández et al. 2016). The higher levodopa bioavailability among women assessed by higher values of the AUC and maximum plasma concentration (Cmax) (Conti et al. 2022) may at least in part explain the higher risk of motor complications among women. As expected, in our sample LEDD was strongly associated with the presence of motor complication, but surprisingly it was not associated with NMF. On this ground, it is possible to hypothesize that dopaminergic mechanisms do not entirely explain the pathogenesis of NMF, which probably involves different pathways. Indeed, even if symptoms fluctuate with dopaminergic treatment, serotonine and norepinephrine denervation, as well as interactions between neurotransmitter systems, may contribute to their diversity (Martínez-Fernández et al. 2016).

Nonetheless, we cannot exclude that some NMS such as sleep, cognition or cardiovascular dysautonomia may also present daily fluctuations not necessarily related to the motor one or to dopaminergic mechanisms. As a matter of fact, this kind difference (daily NMF not dependent on dopaminergic mechanisms) is not always well recognized by PD patients and misinterpretation is possible, also considering that NMS such as cognition and cardiovascular dysfunction might influence each other (Cicero et al. 2019).

We are also aware of the possible limits that should be taken into account in interpreting our findings. First of all, the lack of an official validated Italian version of the NoMoFA questionnaire; for this reason, the questionnaire was administered face-to-face to reduce the risk of misinterpretation. Nevertheless, validation of the Italian questionnaire is required. Second, in agreement with a previous study (Kleiner et al. 2021), we did not consider the cognitive status of PD patients but considering that the questionnaire was administered face-to-face by trained neurologists together with the caregiver (when necessary), we believe that the accuracy and consistency of data were maintained. Moreover, it should be underlined that the NoMoFA questionnaire was originally developed as a patient-reported outcome questionnaire (Kleiner et al. 2021), thus we cannot exclude that the face-to-face interview might has led to a possible operator-dependent bias. Third, as reported above, we cannot exclude that the lack of evaluation in ON phase may have led to an underestimation of NMS in ON phase. Finally, we do not have data about hormone replacement therapy for menopause in women with PD, thus we cannot evaluate the possible effect on motor and non-motor function of estrogen administration.

In conclusion, our study reported the presence of a different gender-related pattern in the occurrence of NMS and NMF in PD patients demonstrating a strong association between female gender and NMF, and suggesting the hypothesis of the existence of different mechanisms involved in these phenomena. The existence of different non-motor phenotype and the role of gender may be relevant in clinical trials and should be considered in finding new treatment strategies that bring us closer to precision medicine.

## Supplementary Information

Below is the link to the electronic supplementary material.

Supplementary file1 (DOCX 14 kb)

Supplementary file2 (DOCX 19 kb)

Supplementary file3 (DOCX 20 kb)

## Data Availability

The data supporting the findings of this study are available on request from the corresponding author.
