# Supplementary material for: Gender differences in non-motor fluctuations in Parkinson’s disease
Source: J Neural Transm (Vienna). 2023 Aug 1;130(10):1249–57. doi: 10.1007/s00702-023-02679-6 (PMC10480257; doi:10.1007/s00702-023-02679-6)
Supplement: Supplementary file 1 — Supplementary file1 (DOCX 14 kb) [file 702_2023_2679_MOESM1_ESM.docx]

**Supplementary table 2** Frequency of non-motor symptoms reported by PD patients and stratified by gender.

| **NMS, n (%)** | **PD sample**  **N=121** | **PD**  **men**  **N=67** | **PD women**  **N=54** | **P value** | **P FDR corrected** |
| --- | --- | --- | --- | --- | --- |
| **1. Lose your train of thought** | 65 (53.7) | 35 (55.2) | 30 (55.6) | 0.71 | 1 |
| **2. Get distracted from completing a task** | 62 (51.2) | 36 (53.7) | 26 (48.1) | 0.54 | 1 |
| **3. Difficulty planning or carrying out an activity** | 47 (38.8) | 24 (35.8) | 23 (42.6) | 0.45 | 1 |
| **4. Confused such that you had difficulty performing simple tasks** | 40 (33.1) | 23 (34.3) | 17 (31.5) | 0.74 | 1 |
| **5. Difficulty finding the right words when speaking** | 87 (71.9) | 54 (80.6) | 33 (61.1) | **0.01** | 0.37 |
| **6. Excessively worried** | 79 (65.3) | 43 (64.2) | 36 (66.7) | 0.77 | 1 |
| **7. Feel scared or threatened** | 20 (16.5) | 12 (17.9) | 8 (14.8) | 0.65 | 1 |
| **8. Feel restless** | 76 (62.8) | 43 (64.2) | 33 (61.1) | 0.73 | 1 |
| **9. Feel hopeless or excessively sad** | 76 (62.8) | 42 (62.7) | 34 (63.0) | 0.97 | 1 |
| **10. Feel lonely or isolated** | 32 (26.4) | 19 (28.4) | 13 (24.1) | 0.59 | 1 |
| **11. See things or people that were not there** | 31 (25.6) | 18 (26.9) | 13 (24.1) | 0.72 | 1 |
| **12. Make poor decisions** | 26 (21.5) | 17 (25.4) | 9 (16.7) | 0.24 | 1 |
| **13. Act quickly without thinking things through** | 31 (25.6) | 22 (32.8) | 9 (16.7) | **0.04** | 0.48 |
| **14. Have a strong uncontrollable urge to do things** | 17 (14.0) | 13 (19.4) | 4 (7.4) | **0.05** | 0.48 |
| **15. Have poor short-term memory** | 61 (50.4) | 36 (53.7) | 25 (46.3) | 0.41 | 1 |
| **16. Have difficulty handling stressful situations** | 48 (39.7) | 30 (44.8) | 18 (33.3) | 0.20 | 1 |
| **17. Lose interest in activities that you previously enjoyed** | 50 (41.3) | 30 (44.8) | 20 (37.0) | 0.39 | 1 |
| **18. Feel sluggish or had low energy levels** | 106 (87.6) | 58 (86.6) | 48 (88.9) | 0.70 | 1 |
| **19. Feel excessively sleepy during the day** | 95 (78.5) | 55 (82.1) | 40 (74.1) | 0.28 | 1 |
| **20. Have painful sensations in your body** | 68 (56.2) | 37 (55.2) | 31 (57.4) | 0.81 | 1 |
| **21. Have strange sensations in your body** | 63 (52.1) | 36 (53.7) | 27 (50.0) | 0.68 | 1 |
| **22. Feel short of breath** | 54 (44.6) | 29 (43.3) | 25 (46.3) | 0.74 | 1 |
| **23. Have problems with vision** | 50 (41.3) | 23 (34.3) | 27 (50.0) | 0.08 | 0.58 |
| **24. Have excessive sweating** | 32 (26.4) | 16 (23.8) | 16 (29.6) | 0.48 | 1 |
| **25. Feel that your heart was racing, had skipped a beat, or was pounding** | 35 (28.9) | 20 (29.8) | 15 (27.8) | 0.80 | 1 |
| **26. Urinate more frequently or felt you had to go to the bathroom urgently** | 86 (71.1) | 51 (76.1) | 35 (64.8) | 0.17 | 1 |
| **27. Have difficulty having a bowel movement** | 85 (70.2) | 52 (77.6) | 33 (61.1) | **0.05** | 0.48 |

Data are given as frequencies (%). PD, Parkinson's Disease; NMS, non-motor symptoms; FDR, false discovery-rate correction.
