# Supplementary material for: Gender differences in non-motor fluctuations in Parkinson’s disease
Source: J Neural Transm (Vienna). 2023 Aug 1;130(10):1249–57. doi: 10.1007/s00702-023-02679-6 (PMC10480257; doi:10.1007/s00702-023-02679-6)
Supplement: Supplementary file 2 — Supplementary file2 (DOCX 19 kb) [file 702_2023_2679_MOESM2_ESM.docx]

**Supplementary table 3** Frequencies of reported domains considering fluctuation status and stratified by gender.

| **DOMAINS, n (%)** | **PD sample**  **N=121** | | | **PD men**  **N=67** | | | **PD women**  **N=54** | | | **p-value**  **men vs women** | | | | | |
| --- | --- | --- | --- | --- | --- | --- | --- | --- | --- | --- | --- | --- | --- | --- | --- |
|  | **NMS,**  **n (%)** | **NMF,**  **n (%)** | **Static NMS, n (%)** | **NMS, n (%)** | **NMF, n (%)** | **Static NMS, n (%)** | **NMS,**  **n (%)** | **NMF,**  **n (%)** | **Static NMS,**  **n (%)** | **P value (NMS)** | **P value (NMS)**  **FDR** | **P value (NMF)** | **P value (NMF) FDR** | **P value (static NMS)** | **P value (static NMS) FDR** |
| **1. Attention** | 82 (67.8) | 33 (40.2) | 49 (59.8) | 46 (68.7) | 15 (32.6) | 31 (67.4) | 36 (66.6) | 18 (50) | 18 (50) | 0.81 | 0.84 | 0.17 | 0.11 | 0.14 | **0.04** |
| **2. Language** | 88 (72.7) | 31 (35.2) | 57 (64.8) | 55 (82.1) | 15 (27.3) | 40 (72.7) | 33 (61.1) | 16 (48.5) | 17 (51.5) | 0.01 | 0.12 | 0.36 | 0.19 | **0.001** | **0.006** |
| **3. Memory** | 63 (52.1) | 11 (17.5) | 52 (82.5) | 38 (56.7) | 3 (7.9) | 35 (92.1) | 25 (46.3) | 8 (32) | 17 (68) | 0.25 | 0.6 | **0.05** | **0.05** | **0.02** | **0.016** |
| **4. Depression/anxiety** | 99 (81.8) | 39 (39.4) | 60 (60.6) | 54 (80.6) | 13 (24.1) | 41 (75.9) | 45 (83.3) | 26 (57.8) | 19 (42.2) | 0.70 | 0.84 | **0.0007** | **0.005** | **0.004** | **0.008** |
| **5. Impulsivity** | 72 (59.5) | 21 (29.2) | 51 (70.8) | 46 (68.6) | 10 (21.7) | 36 (78.3) | 26 (48.2) | 11 (42.3) | 15 (57.7) | 0.02 | 0.12 | 0.43 | .**020** | **0.004** | **0.008** |
| **6. Apathy** | 52 (43.0) | 6 (11.5) | 46 (88.4) | 32 (47.8) | 0 (0) | 32 (100) | 20 (37.0) | 6 (30) | 14 (70) | 0.23 | 0.6 | **0.02** | **0.03** | **0.01** | **0.01** |
| **7. Hallucination/perception** | 59 (48.7) | 10 (16.9) | 49 (83.1) | 30 (44.8) | 6 (20) | 24 (80) | 29 (53.7) | 4 (13.8) | 25 (86.2) | 0.17 | 0.6 | 0.75 | 0.30 | 0.24 | 0.06 |
| **8. Sleep/fatigue** | 116 (95.8) | 61 (52.6) | 55 (47.4) | 64 (95.3) | 23 (35.9) | 41 (64.1) | 52 (96.3) | 38 (73.1) | 14 (26.9) | 0.83 | 0.84 | **0.0008** | **0.005** | **0.0001** | **0.002** |
| **9. Dysautonomia** | 114 (94.2) | 19 (16.7) | 95 (83.3) | 63 (94.1) | 5 (7.9) | 58 (92.1) | 51 (94.4) | 14 (27.5) | 37 (72.5) | 0.92 | 0.84 | **0.005** | **0.01** | **0.02** | **0.01** |
| **10. Miscellaneous** | 98 (81.0) | 30 (30.6) | 68 (69.4) | 54 (80.6) | 11 (20.4) | 43 (79.6) | 44 (81.5) | 19 (43.2) | 25 (56.8) | 0.90 | 0.84 | **0.02** | **0.03** | **0.05** | **0.03** |
| **11. Cognitive*** | 105 (86.8) | 52 (49.5) | 53 (50.5) | 61 (91.0) | 24 (39.4) | 37 (60.6) | 44 (81.4) | 28 (36.6) | 16 (36.4) | 0.12 | 0.56 | **0.07** | **0.064** | **0.004** | **0.008** |

Data are given as frequencies (%). PD, Parkinson's Disease; NMS, non-motor symptoms; NMF, non-motor fluctuations; FDR, false discovery-rate correction.

*considering attention, language and memory as one domain
