# Supplementary material for: Gender differences in non-motor fluctuations in Parkinson’s disease
Source: J Neural Transm (Vienna). 2023 Aug 1;130(10):1249–57. doi: 10.1007/s00702-023-02679-6 (PMC10480257; doi:10.1007/s00702-023-02679-6)
Supplement: Supplementary file 3 — Supplementary file3 (DOCX 20 kb) [file 702_2023_2679_MOESM3_ESM.docx]

**Supplementary table 1** Clinical, demographic characteristics and motor complications: univariate and multivariate analysis.

|  | ***Univariate analysis*** | | | ***Multivariate analysis*** | | |
| --- | --- | --- | --- | --- | --- | --- |
|  | **OR** | **95%CI** | **P value** | **OR** | **95%CI** | **P value** |
| **Gender** |  |  |  |  |  |  |
| **Male (Ref)** | 1 | / | / | 1 | / | **/** |
| **Female** | 2.32 | 1.10-4.87 | **0.03** | 3.48 | 1.45-8.39 | **0.004** |
| **Age (years)** | 0.97 | 0.93-1.01 | 0.1 | 0.95 | 0.90-0.99 | **0.03** |
| **Age at onset** | 0.89 | 0.81-0.96 | **0.006** | / | / | / |
| **Disease duration (years)** | 1.13 | 1.03-1.24 | **0.005** | 1.05 | 0.95-1.18 | 0.3 |
| **Hoehn – Yahr stage** | 1.46 | 0.89-2.38 | 0.1 | 1.43 | 0.78-2.64 | 0.2 |
| **UPDRS-ME score (OFF)** | 1.00 | 0.97-1.03 | 0.7 | / | / | / |
| **LEDD ≤ 500 mg (Ref)** | 1 | / | / | 1 | / | / |
| **LEDD > 500 mg** | 4.97 | 2.27-10.85 | **<0.0001** | 5.12 | 1.91-13.7 | **0.001** |

Multivariate logistic regression analysis adjusted for age and gender. UPDRS-ME, Unified Parkinson's Disease Rating Scale-Motor Exam; LEDD, Levodopa equivalent daily dose.
